# Fermented Stevia Improves Alcohol Poisoning Symptoms Associated with Changes in Mouse Gut Microbiota

**DOI:** 10.3390/nu15173708

**Published:** 2023-08-24

**Authors:** Qingmiao Ma, Masafumi Noda, Narandalai Danshiitsoodol, Masanori Sugiyama

**Affiliations:** Department of Probiotic Science for Preventive Medicine, Graduate School of Biomedical and Health Sciences, Hiroshima University, Minami-ku, Hiroshima 734-8551, Japan; ma1202qingmiao@gmail.com (Q.M.); bel@hiroshima-u.ac.jp (M.N.); naraa@hiroshima-u.ac.jp (N.D.)

**Keywords:** lactic acid bacteria, medicinal herbs, stevia, dysbiosis, gut microbiota

## Abstract

We previously found that the continuous feeding of ethanol caused mice dysbiosis, in which the cecal microbiota were significantly altered, as compared with those in the non-feeding control group, especially in some bacterial genera involved in gut inflammation. In the present study, we have found that the fermented extract of stevia (*Stevia rebaudiana*) leaves with plant-derived lactic acid bacteria (LABs), *Pediococcus pentosaceus* LY45, improves the trimethylamine (TMA) productivity of cecal content, which can be used as an indicator of dysbiosis. The following animal experiment also shows that the LY45-fermented stevia extract represses the typical increase in serum aspartate aminotransferase (AST) and alanine aminotransferase (ALT) levels, which decreased from 1106 to 210 IU/L (*p* < 0.05) and from 591 to 100 IU/L (*p* < 0.05), respectively, together with the simultaneously latent TMA productivity (from 1356 to 745 μM, *p* < 0.05) of cecal content in the ethanol-fed mice. The microbiota analyses have shown that the observed increased alterations in pro-inflammatory genera putative *SMB53* (family Clostridiaceae) and *Dorea* are restored by the fermented stevia extract. Our result indicates that the preliminary bioconversion of herbal medicinal precursors by fermentation with safe microorganisms like LABs is expected to be a hopeful method of producing specific metabolites that may contribute to the reconstruction of gut microbiota.

## 1. Introduction

Medicinal herbs contain important molecules with therapeutic potential [1], and some of them have been used as components in Chinese medicine. Because the focus of Chinese medicine is not just on illness, but also on the patient as an individual, some medicinal herbs have been used widely, not only in Asian countries, but also in the United States of America (USA) and Europe [2,3]. In general, the biologically active substances synthesized in plants are stored as glycoside forms of aglycones to improve their stability and water solubility; however, the biological activity of glycosides is quite a bit lower, due to their low bioavailability compared with that of corresponding aglycones [4]. After ingestion, aglycones have been reported to be generally released from glycosyl residues by gut microbiota [5,6]. The plant-derived substances also undergo biotransformation by the gut microbiota, such as deglycosylation, dehydroxylation, reduction, C-ring cleavage, and demethylation [7], suggesting that the microorganisms play a significant role in displaying the maximum effect of medicinal herbs by conversion of the substances through fermentation. Thus, the application of the fermented medicinal herbal extract is an attractive approach to improve the efficacy of those herbal medicines.

Recently, gut microbiota have been reported to be associated with human health and diseases [8,9], such as obesity [10], liver diseases [11], and neuropsychiatric diseases [12]. Those reports indicate that dysbiosis, which is regarded as a disturbance of gut microbial homeostasis, may be responsible for multiple disorders [13,14]. Our previous study showed that the administration of live LAB cells can restore not only the fluctuations in the cecal microbiota and metabolites related to putrefaction, but also alcohol poisoning symptoms in the ethanol-fed-mouse model [15]. In that model, the abundance of a putative order RF32, which has been reported to positively correlate with the serum trimethylamine *N*-oxide (TMAO) level [16], was remarkably increased. Dietary substrates, including choline, carnitine, and betaine, are partially used by gut microorganisms to form trimethylamine (TMA), which is subsequently enzyme-converted to TMAO in the liver [17,18]. TMAO has been increasingly recognized as a risk marker of cardiometabolic and hepatic diseases, as well as other chronic diseases [19,20]; therefore, suppressing microbial TMA production by the alteration of disturbed gut microbiota seems to be an ideal preventive approach against those diseases.

Although our previous studies have shown that biologically active aglycones are released from their corresponding glycosides, and some bioactive substances are newly generated in medicinal herbal extracts fermented with plant-derived lactic acid bacteria (LABs) [21], their efficacy in modulating gut microbiota has not been dealt with yet. Therefore, in the present study, we have focused on whether the dysbiosis will be repaired by the application of the fermented medicinal herbal extract, using the level of TMA production as an indicator.

## 2. Materials and Methods

### 2.1. Preparation of the Medicinal Herbal Extract

The medicinal herbs were purchased from Kojima Kampo (Osaka, Japan) in their cut, dried forms. Twenty kinds of medicinal herbs were used in the present study, and the IDs of those are as follows: 1, Glycyrrhizae Radix (*Glycyrrhiza glabra* Linné); 2, leaves of *Stevia rebaudiana*; 3, Saussureae Radix; 4, roots of *Glycyrrhiza uralensis* Fisher; 5, Scutellariae Radix; 6, Armeniacae Semen; 7, Sesami Semen; 8, Lili Bulbus; 9, Epimedii Herba; 10, Cnidii Rhizoma; 11, Polygoni Multiflori Radix; 12, Eriobotryae Folium; 13, Rhei Rhizoma; 14, Artemisiae Folium; 15, Atractylodis Lanceae Rhizoma; 16, Moutan Cortex; 17, Paeoniae Radix; 18, Persicae Semen; 19, Gardeniae Fructus; and 20, Lycium Fructus. Small pieces of herbs were suspended in distilled water to a final concentration of 5% (*w*/*v*) and then heat-treated at 105 °C for 30 min. The herbal debris was removed from the extracts by centrifugation, and the obtained supernatants were adjusted to pH 7.0 by adding sodium hydroxide (FUJIFILM Wako Pure Chemical Corporation, Osaka, Japan) solution. After sterilization at 120 °C for 20 min, the extracts were used as cultivation media of the LABs.

### 2.2. Bacterial Strains and Culture Conditions

The bacterial strains used in the present study are listed in Table 1. For seed cultivation, LAB strains were cultivated in de Man, Rogosa, and Sharpe (MRS) broth (Merck KGaA, Darmstadt, Germany), at an appropriate temperature (28–45 °C), as the standing cultivation. Then, each seed culture was inoculated into the prepared medicinal herbal extract at 1% (*v*/*v*) and incubated at the same temperature for 24 h in the same culture method. After cultivation, each culture was sterilized at 120 °C for 20 min, followed by centrifugation to remove the bacterial cell debris. The obtained supernatant fluid was used as the fermented medicinal herbal extract. Alternatively, the supernatant fluid collected from the medicinal herbal extract without inoculation of any microbes was used as the unfermented one.

### 2.3. Establishment of an Alcohol-Poisoning Mouse Model

The animal experiments in the present study were carried out in accordance with the experimental procedures approved by the Committee of Research Facilities for Laboratory Animal Science of Hiroshima University (Permit Number: A16-9-2). All animal experiments were conducted in accordance with the “Guidelines for the Care and Use of Laboratory Animals” of Hiroshima University.

Seven-week-old age-specific pathogen-free (SPF) male C57BL/6JJmsSlc mice (purchased from Shimizu Laboratory Supplies, Co., Ltd., Kyoto, Japan) were employed in the present study. All mice were reared in a plastic cage under conditions of 20–26 °C temperature, 40–60% humidity, and a 12 h light/12 h dark cycle. The mice were maintained with free access to drinking water and regular rodent feed (MF diet, Oriental Yeast, Co., Ltd., Tokyo, Japan) during a one-week familiarity period. The diet was then changed to an L10016 diet (Research Diet, New Brunswick, NJ, USA) prepared from Pre-Mix L10016A (Research Diet), in accordance with the manufacturer’s instructions, with a slight modification. Briefly, 7.5% (*v*/*v*) ethanol (SAKURAO Brewery and Distillery Co., Ltd., Hiroshima, Japan) at a final concentration and 118.27 g/L maltodextrin 42 (Research Diet) were added to Pre-Mix L10016A to prepare L10016 and L10015, which is a non-ethanol-containing diet used in the non-ethanol-fed group (negative control), respectively. Because both diets were liquid and contained sufficient water, all mice were kept without any additional drinking water.

### 2.4. In Vitro TMA Production Inhibitory Assay

After the two-week rearing period, the mice were euthanized via the inhalation of anesthesia with isoflurane, and the cecum was collected from the alcohol-poisoning-model mice and the non-ethanol-feeding mice. A portion of the cecal contents was inoculated into a modified Gifu Anaerobic Broth (GAM) media (Nissui Pharmaceutical Co., Ltd., Tokyo, Japan) and anaerobically incubated at 37 °C for 24 h. The culture was then mixed with the fermented medicinal herbal extract at a 1:4 ratio and further incubated under the same conditions for 24 h. The LAB cells were removed from the cultured mixture, and the TMA concentration of the supernatant was calculated using a modified Dyer method [28,29]. Briefly, 0.2 mL of 1:3 diluted neutral formalin (FUJIFILM Wako Pure Chemical Corporation), 2 mL of dehydrated toluene (FUJIFILM Wako Pure Chemical Corporation), and 0.6 mL of 25% (*w*/*v*) potassium hydroxide (FUJIFILM Wako Pure Chemical Corporation) solution were added to 1 mL of the supernatant in a sequenced order, and the mixture was vortexed vigorously for 1 min. After standing for 5 min at room temperature, the toluene layer was collected and dehydrated with sodium sulfate anhydride (FUJIFILM Wako Pure Chemical Corporation). Finally, a given toluene extract was mixed with the same volume of 0.02% (*w*/*v*) picric acid (FUJIFILM Wako Pure Chemical Corporation) solution, and the concentration of the generated picrate was calculated spectrophotometrically at 450 nm based on a standard curve, prepared using solutions of TMA from 67.2 μM to 4.3 mM.

### 2.5. Animal Experiments to Evaluate the Impact of the Fermented Stevia Extracts on Alcohol-Poisoning-Model Mice

After being fed with a regular rodent diet in the familiarity period, the male SPF C57BL/6JJmsSlc mice (seven weeks of age) were divided into four experimental groups (five mice each) as follows: a group fed only an L10015 diet (without ethanol, negative control group, NC); a group fed only an L10016 diet (ethanol-containing, positive control group, PC); a group fed an L10016 diet with the simultaneous administration of unfermented stevia extract (unfermented group, Unf); and a group fed an L10016 diet with the simultaneous administration of fermented stevia extract (fermented group, Fer). Each mouse was weighed prior to the experimental period and was distinguished by different-colored markings on their tails using Animal Marker felt pens (Fuchigami Kikai Co., Ltd., Kyoto, Japan). A 100 μL aliquot of the stevia extract samples was administered every day during the experimental period using an oral feeding needle to the Unf and Fer groups. The same volume of sterile water was administered to the PC and NC groups instead of the extract.

After the experimental period, the mice were euthanized with isoflurane (FUJIFILM Wako Pure Chemical Corporation), and blood and cecum were collected from each mouse. Serum aspartate aminotransferase (AST), alanine aminotransferase (ALT), alkaline phosphatase (ALP), lactate dehydrogenase (LDH), cholinesterase (ChE), and total cholesterol (T-Cho) levels were measured by Oriental Yeast Co., Ltd., Tokyo, Japan. The TMA production abilities of each cecal content were evaluated and compared, as described above.

### 2.6. Analysis of Cecal Microbiota

The 16S rRNA-based microbiota analysis of the cecal contents was performed at Bioengineering Lab. Co., Ltd. (Kanagawa, Japan) by a 300-bp read length paired-end protocol using the Illumina MiSeq sequence platform and MiSeq Reagent Kit v3 (Illumina Inc., San Diego, CA, USA). Briefly, the total DNA from the cecal contents of each group was extracted and purified using an MPure-12 Automated Nucleic Acid Purification System and an MPure Bacterial DNA Extraction Kit (MP Biomedicals, Santa Ana, CA, USA). The V3–V4 region of the 16S rRNA genes was amplified with the primer sets 5′-ACACTCTTTCCCTACACGACGCTCTTCCGATCT-N_n_-CCTACGGGNGGCWGCAG-3′ and 5′-GTGACTGGAGTTCAGACGTGTGCTCTTCCGATCT-N_n_-GACTACHVGGGTATCTAATCC-3′ (the N_n_ indicates that each sequence consists of random nucleotides with random bases) using ExTaq HS (Takara Bio Inc., Shiga, Japan) under the following conditions: 2 min at 94 °C, followed by 20–25 cycles of 30 s at 94 °C, 30 s at 55 °C, and 30 s at 72 °C, and, finally, a 5 min extension period at 72 °C. The amplified fragments were purified using an Agencourt AMPure XP kit (Beckman Coulter Inc., Brea, CA, USA), in accordance with the manufacturer’s protocol. A 2 μL aliquot of each resultant solution containing purified fragments was further used as a template for a second PCR reaction with the primer sets 5′-AATGATACGGCGACCACCGAGATCTACAC-X_8_-ACACTCTTTCCCTACACGACGC-3′ and 5′-CAAGCAGAAGACGGCATACGAGAT-X_8_-GTGACTGGAGTTCAGACGTGTG-3′ (the X_8_ indicates index sequences designed to identify each sample in the system) under the following conditions: 2 min at 94 °C, followed by 20–25 cycles of 30 s at 94 °C, 30 s at 55 °C, and 30 s at 72 °C, and, finally, a 5 min extension period at 72 °C. The amplified samples were also purified, as described above, and then conducted to the analytical instrument. The sequence analysis and taxonomic assignment were performed using the Quantitative Insights into Microbial Ecology (QIIME) 2.0 pipeline [30].

## 3. Results

### 3.1. Effect on the TMA Productivity of the Medicinal Herbal Extract

**Figure 1:**
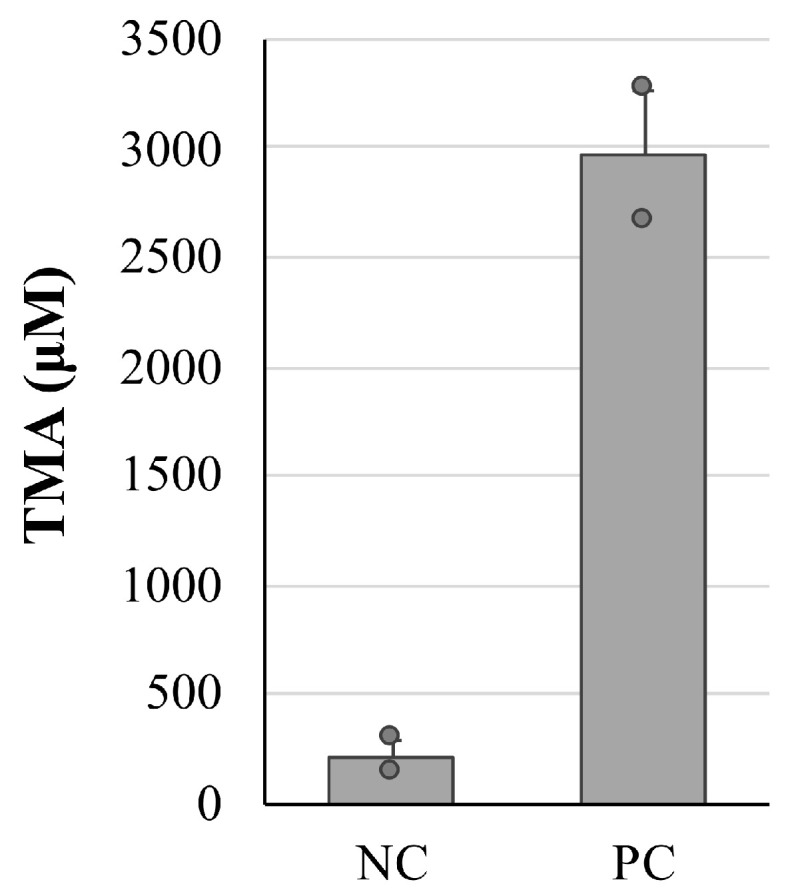
Differences in the trimethylamine (TMA) concentrations in the cultured broth of cecum samples. Cecum samples were collected from mice without ethanol feeding (negative control group, NC) and ethanol-fed mice (positive control group, PC). The values are indicated as the mean ± standard error (S.E.).

In our previous study, the microbiota analysis performed on the cecal samples collected from dysbiosis-induced mice by ethanol feeding showed that the relative abundance of putative RF32 order, which has been reported to correlate with the serum TMAO level [16], was increased by ethanol intake [15]. In the present study, we first tried to compare the TMA concentration of the cecum samples in ethanol-fed mice (PC group) with a negative control group of mice (NC group; without ethanol feeding). However, the TMA concentrations could not be determined by spectrophotometric assay [28,29], which may have been due to its measurement limit. Thus, we measured and compared the TMA production in the cultured broth of the cecum samples (Figure 1). The results showed that ethanol feeding altered the cecal contents to have latent TMA productivity (about 15-fold). Thus, we used the TMA production level as an indicator of cecal dysbiosis.

**Figure 2:**
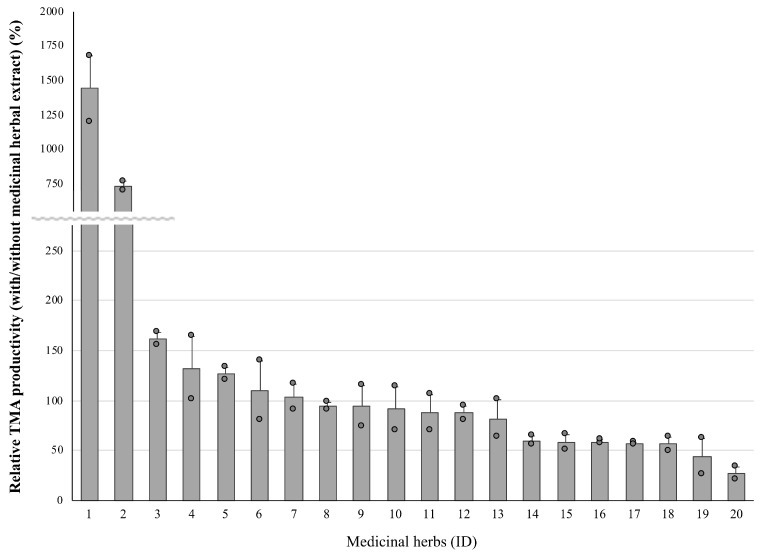
A comparison of the relative latent TMA productivities of cecum samples collected from ethanol-fed mice in the presence of unfermented medicinal herbal extracts. IDs of the medicinal herbs are summarized in Section 2. The values are indicated as the mean ± S.E. (*n* = 2).

Next, whether or not the medicinal herbal extract altered the latent TMA productivity was confirmed by using 20 kinds of extract (Figure 2). When compared with the without-extract sample, some of the herbal extracts reduced the TMA production, especially in Gardeniae Fructus (ID 19) and Lycium Fructus (ID 20). On the other hand, the extracts from Glycyrrhizae Radix (ID 1) and stevia (leaves of *Stevia rebaudiana*, ID 2) showed marked TMA inducibility.

### 3.2. Inhibitory Activity Alteration of Medicinal Herbal Extract before and after LAB Fermentation

**Figure 3:**
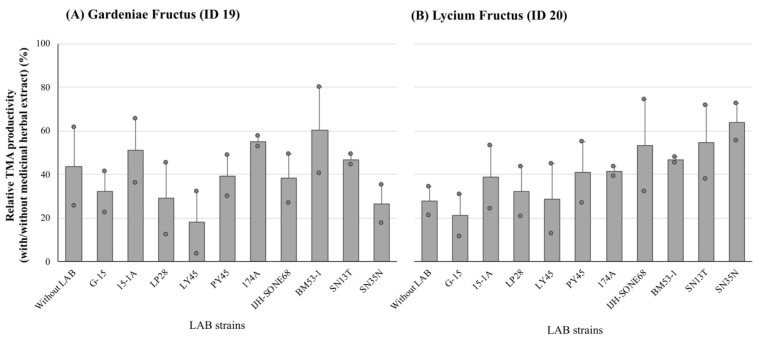
The relative latent TMA productivities of the cecum samples collected from ethanol-fed mice in the presence of lactic acid bacteria (LAB)-fermented Gardeniae Fructus (**A**) and Lycium Fructus (**B**) extracts. The values are indicated as the mean ± S.E. (*n* = 2).

Among the tested samples, the extracts from Gardeniae Fructus and Lycium Fructus were used for further study to compare the anti-TMA production activity of the herbal extracts before and after LAB fermentation. In this study, 10 kinds of LAB strains, which were isolated in our research group and expected to be useful and helpful for human health care (Table 1), were used to ferment these herbal extracts. As compared with the unfermented extract of Gardeniae Fructus, the fermented extracts by LP28, LY45, and SN35N strains showed relatively higher reduction abilities against TMA production (Figure 3A), whereas, in Lycium Fructus, the anti-TMA activity of the fermented extracts tended to be reduced (Figure 3B). Based on these results, these three strains were used for further analysis.

**Figure 4:**
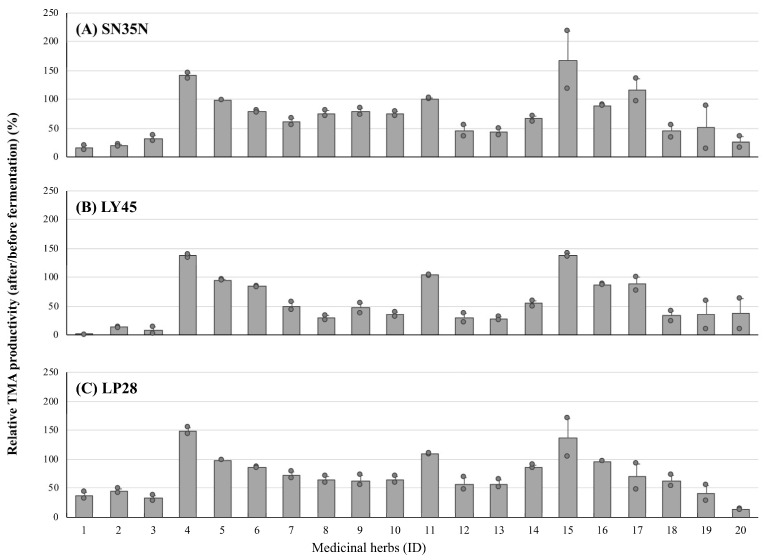
The changes in TMA productivities of the cecum samples collected from ethanol-fed mice in the presence of medicinal herbal extracts fermented with *L. plantarum* SN35N (**A**), *P. pentosaceus* LY45 (**B**), and *P. pentosaceus* LP28 (**C**). IDs of the medicinal herbs are summarized in Section 2. The values are indicated as the mean ± S.E. (*n* =2).

To further confirm the effect of LAB fermentation on the herbal extracts, we again compared the inhibitory activity against the TMA production of all of the tested herbal extracts before and after LAB fermentation (Figure 4). Among the three strains, the activity seemed to be improved in the LY45-fermented extracts. Interestingly, LAB fermentation markedly decreased the TMA induction observed in the unfermented extracts from Glycyrrhizae Radix and stevia, especially by LY45 fermentation. Because Glycyrrhizae Radix contains a large amount of glycyrrhizin, which has been recognized to counteract antihypertensive medications by its pseudo-aldosterone effect [31,32], the LY45-fermented stevia extract was used for the additional in vivo verification experiment.

### 3.3. Improving the Effect of Fermented Stevia Extracts on Alcohol-Poisoning-Model Mice

**Figure 5:**
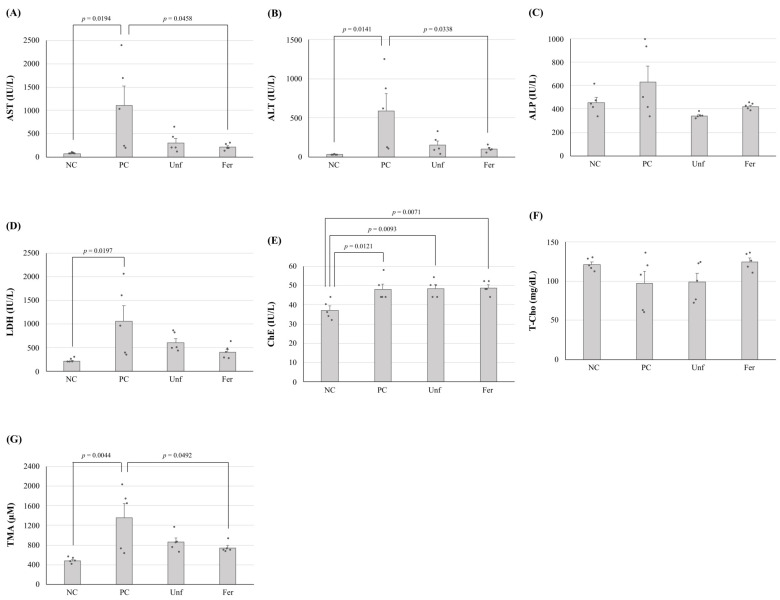
Differences in the serum aspartate aminotransferase (AST) (**A**), alanine aminotransferase (ALT) (**B**), alkaline phosphatase (ALP) (**C**), lactate dehydrogenase (LDH) (**D**), cholinesterase (ChE) (**E**), total cholesterol (T-Cho) (**F**) levels, and latent TMA productivities of the cecum samples (**G**) between each experimental group. Samples were collected from mice without ethanol feeding (NC), ethanol-fed mice (PC), and ethanol and unfermented (Unf) or fermented (Fer) stevia extract-fed mice. The values are indicated as the mean ± S.E. (*n* = 5). Statistical analyses were performed using the Tukey–Kramer multiple comparison test.

The LY45-fermented or unfermented stevia extracts were orally administrated, and the differences in their effect on improving alcohol-poisoning-model mice before and after fermentation were evaluated (Figure 5). The improvement observed in hepatic parameters AST and ALT, which significantly (*p* < 0.05) increased in the ethanol-fed group (positive control, PC), as compared with the negative control group (NC, without ethanol-feeding), were not significant in the unfermented group (Unf), but were significant (*p* < 0.05) in the fermented group (Fer) (Figure 5A,B). Similar results were observed in LDH, but there were no significant differences among the treatment groups (Figure 5D). When the latent TMA productivities of the cecal contents collected from each mouse were compared, the increased TMA production observed in the PC group was repressed in the Fer group, with statistical significance (*p* < 0.05, Figure 5G). In the other items, there were no noticeable effects caused by the treatments.

### 3.4. Alterations of Cecal Microbiota

**Figure 6:**
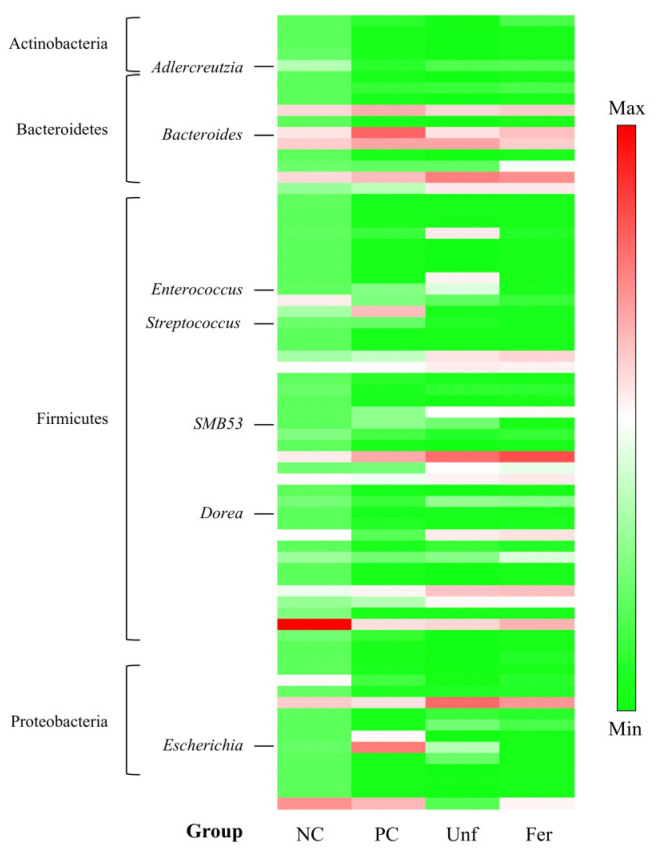
The cecal microbiota in each group were determined using the V3–V4 region in 16S rDNA and indicated by the standardized relative abundance ratio. Denser green and red indicate lower and higher amounts, respectively. The experimental content of each group is the same as that in Figure 5.

The cecal microbiota of the alcohol-poisoning-model mice treated with the samples were also compared (Figure 6 and Table 2). We focused on the increased (which became detectable or increased by four times or more) or decreased (which became non-detectable or decreased by four times or more) categories after ethanol feeding. The abundance of some genera (*Allobaculum*, *Sutterella*, *Lactobacillus*, *Adlercreutzia*, *Anaerofustis*, and *Prevotella*) was remarkably decreased by the administration of ethanol; however, of those, only *Adlercreutzia* was found to be restored by taking the fermented (Fer) or unfermented (Unf) stevia extracts. On the other hand, although the ratio of *Escherichia*, *Bacteroides*, *Enterococcus*, *SMB53* (putative), and *Dorea* genera were notably increased by ethanol feeding, these phenomena disappeared with the administration of the fermented stevia extract. The increased abundance of *Lactococcus*, *Clostridium*, and *Staphylococcus* also observed in the ethanol-fed group (PC) was not restored by the extracts. Only in the *Streptococcus* genus, which also increased in the PC group, did it remain unrestored in the Fer group, but not in the Unf group. The unfermented stevia extract also improved the *Bacteroides* alteration.

## 4. Discussion

Our previous study demonstrated that live LAB cells (SN13T strain) are significant for improving the intestinal microbiota balance and protecting the host from dysbiosis in mice suffering from fatal alcohol poisoning symptoms [15]. The disturbed cecal microbiota of the model mice were altered to produce TMA potentially. TMAOs, widely known as risk markers for some chronic diseases, are enzymatically converted in the liver from TMA [17,18,19,20], metabolically generated by the gut microbiota from choline, which plays an important role, not only in body structural integrity, but also in brain and nervous system functions [33]. The acceleration of TMA bioconversion from choline by imbalanced gut microbiota may lead to choline deficiency, which may cause liver damage and hepatosteatosis, in addition to serum TMAO increase, resulting in a high risk of hepatic inflammation [33,34,35,36]. Our results indicate that the cecal microbiota with dysbiosis induced by ethanol feeding carry those latent risks.

The present study has revealed that LY45-fermented stevia extract can repress TMA production with statistical significance during cecal microbiota cultivation in both in vitro and in vivo experiments. Interestingly, the administration of unfermented stevia extract was moderately effective at improving AST, ALT, and TMA values in animal experiments, whereas an in vitro assay showed that the same unfermented extract considerably enhanced the TMA productivity (Figure 2). This may be due to the fact that, during gastrointestinal processing, the constituents of the unfermented stevia extract will have undergone digestion, degradation, and metabolism reactions, which contribute to the reduction in TMA productivity. The resultant metabolites are partially similar to those of the LY45-fermented extract. This hypothesis presumes that the observed improvement effects were caused, not by the substances produced by the LY45 strain itself, but by the metabolites derived from the components contained in the stevia extract generated by microorganisms during the fermentation process.

Gut microbiota have been reported to play an important role in the bioconversion of the precursor contained in herbal medicines into bioactive molecules [37,38,39]; thus, the medicinal effect of herbal extracts may depend on the microbiota composition. Recently, it has been reported that the anti-cancer therapeutic efficacy of immune checkpoint inhibitors targeting programmed cell death protein 1 (PD-1), such as the human IgG4 monoclonal antibody Nivolumab, was affected by the composition of the resident gut microbiota [40]. In this case, several bacterial species are predicted to play key roles in the efficacy by increasing tumor-specific CD8^+^ T cells and the T-helper type 1 cell (Th1)/regulatory T cell (T_reg_) ratio.

In the present study, the animal experiment was performed with inbred SPF mice; thus, the gut conditions of all of the mice were rather similar to one another. Although the unfermented stevia extract also showed an improving effect on the ethanol-fed model mice, having no resident gut bacteria that can convert the precursor into an active form may result in worsening the symptoms, rather than an ineffectual result. The preliminary bioconversion of herbal medicinal precursors or natural products by fermentation with safe microorganisms, such as probiotics, including LABs, is expected to be a promising method to avoid problems based on individual differences in response to medicinal compounds needing to undergo the bioconversion process.

Different from the previous study, in which the live SN13T cells may improve dysbiosis through a probiotic effect [15], the LY45-fermented stevia extract did not contain living cells. This indicates that substances besides live probiotic cells can play a role in improving the gut microbiota. In fact, the present study showed that the administration of the fermented stevia extract restored the abundance of *Escherichia* and *Enterococcus* genera in the alcohol-fed model mice. Non-pathogenic *Escherichia coli* is the predominant species in the genus *Escherichia* and is recognized as one of the resident gut microbiota contributing to vitamin K production and protection against external pathogens in healthy human adults [41]. The genus *Enterococcus* is also known to be a normal constituent of the natural microbiota, and some strains of enterococci have been used in the treatment of various diseases as probiotic therapies [42,43,44]. Although further studies to clarify what kinds of substances improve the observed conditions are now in progress, it is hard to predict whether sugars, vitamins, and minerals, which can promote the growth of an indefinite number of bacteria, would remain after fermentation, with LAB strains having multiple auxotrophies [45]. Therefore, specific metabolites produced during fermentation may contribute to the reconstruction of the gut microbiota.

From the microbiota analysis, the observed increased alterations in genera putative *SMB53* (family Clostridiaceae) and *Dorea* were also restored by the fermented stevia extract. The *SMB53* genus has been reported to be a pro-inflammatory group in the intestinal tract and correlates with TMA and TMAO levels, obesity, type II diabetes, and hepatosteatosis [46,47,48,49]. In addition, the *SMB53* genus is particularly abundant in hepatocellular carcinoma-bearing epigenetic instability model *Trim28^hep–/–^* mice [50]. Although we have not performed animal experiments to evaluate those disorders, because the fermented stevia extract had an improving effect on TMA production, the extract is expected to be effective on inflammatory disorders related to the genus *SMB53*. In the *Dorea* genus, although two species—*Dorea longicatena*, which has been reported to be a possible marker to predict hepatic steatosis [51,52], and *Dorea formicigenerans*—may correlate with the poor conditions of patients suffering with cirrhosis and fatty liver disease [53,54], different species of *Dorea* have been reported to be associated with different conditions in obese non-alcoholic fatty liver disease (NAFLD) patients [55,56]. Those reports suggest that various potent properties associated with gut microbiota depend, not on the genus, but on the species or strain; however, the *Dorea* species observed here in increased numbers in the ethanol-fed mice may be an undesirable species for health promotion. Thus, further analysis of the determination of those *Dorea* species will give new insights into the treatment of gut dysbiosis.

In conclusion, the present study has demonstrated that *Pediococcus pentosaceus* LY45 fermentation of the stevia leaf extract can enhance its ability to improve serum AST and ALT levels and latent TMA productivity of cecal content, which significantly increased in the ethanol-fed mice. Our results suggest that the LY45-fermented stevia extract may be a useful technology to produce substrates that reconstruct the gut microbiota. Unfortunately, it has still not been determined what kinds of substrate were produced after the fermentation of the extract. However, our results have also indicated the significant potential of the fermentation of a medicinal herb with LABs to generate active compounds for preventing and improving in gut dysbiosis.

## Figures and Tables

**Table 1:** LAB strains used in this study.

Species	Strain	Cultivation Temperature	Notes	References
*Enterococcus avium*	G-15	37 °C	γ-Aminobutyric acid (GABA) production	[22]
*Enterococcus mundtii*	15-1A	37 °C	Bacteriocin production	[22]
*Pediococcus pentosaceus*	LP28	28 °C	Exopolysaccharide (EPS) production, Anti-obesity	[22]
	LY45	45 °C	Thermophilic, EPS production	[23]
*Lactobacillus amylovorus*	PY45	45 °C	Thermophilic, EPS production	[23]
*Lactobacillus brevis*	174A	28 °C	Bacteriocin production	[24]
*Lactobacillus paracasei*	IJH-SONE68	28 °C	EPS production, Anti-inflammation	[22,25]
*Lactobacillus reuteri*	BM53-1	28 °C	Anti-biofilm	[26]
*Lactobacillus plantarum*	SN13T	28 °C	Anti-constipation, Improving liver function	[15,22]
	SN35N	28 °C	EPS production, Anti-virus infection	[22,27]

**Table 2:** A summary of changes in cecal microbiota composition.

	Phylum	Restore ^3^		Class	Restore		Order	Restore	
		Fer	Unf		Fer	Unf		Fer	Unf
Increased ^1^				Gammaproteobacteria	†		Enterobacteriales		
				Bacilli			Bacillales		
Decreased ^2^	Actinobacteria	†	†	Erysipelotrichi			Erysipelotrichales		
				Verrucomicrobiae			Burkholderiales		
				Betaproteobacteria			Coriobacteriales	†	†
				Coriobacteriia					
				Deltaproteobacteria	†	†			
	**Family**	**Restore**		**Genus**	**Restore**				
		**Fer**	**Unf**		**Fer**	**Unf**			
Increased ^1^	Enterobacteriaceae	†		*Escherichia*	†				
	Enterococcaceae	†		*Bacteroides*	†	†			
	Streptococcaceae			*Enterococcus*	†				
	Clostridiaceae			*Lactococcus*					
	Staphylococcaceae			*Clostridium*					
	Rikenellaceae			*Staphylococcus*					
	Bacteroidaceae	†	†	*SMB53*	†				
	Mogibacteriaceae	†		*Streptococcus*		†			
				*Dorea*	†				
Decreased ^2^	Erysipelotrichaceae			*Allobaculum*					
	Alcaligenaceae			*Sutterella*					
	Lactobacillaceae			*Lactobacillus*					
	Desulfovibrionaceae	†	†	*Adlercreutzia*	†	†			
	Coriobacteriaceae	†	†	*Anaerofustis*					
	Christensenellaceae	†	†	*Prevotella*					
	Eubacteriaceae								
	Prevotellaceae								

The bacteria that belong to the categories listed in the table increased or decreased in their proportions by ethanol-feeding when compared with the non-ethanol-diet control group. ^1^ “Increased” means the categories that became detectable or that increased by 4 times or more only after ethanol-feeding. ^2^ “Decreased” means the categories that became non-detectable or that decreased by 4 times or more only after ethanol-feeding. ^3^ “Restore” means the changed proportions were restored by taking fermented (Fer) or unfermented (Unf) stevia extract and are indicated by dagger (†).

## Data Availability

The data presented in the study are available in article.

## References

[1] Atanasov A.G., Waltenberger B., Pferschy-Wenzig E.M., Linder T., Wawrosch C., Uhrin P., Temml V., Wang L., Schwaiger S., Heiss E.H. (2015). Discovery and resupply of pharmacologically active plant-derived natural products: A review. Biotechnol. Adv..

[2] Lee K.H. (2000). Research and future trends in the pharmaceutical development of medicinal herbs from Chinese medicine. Public Health Nutr..

[3] Foster S., Tyler V.E. (1999). Tyler’s Honest Herbal: A Sensible Guide to the Use of Herbs and Related Remedies.

[4] Lee N.K., Paik H.D. (2017). Bioconversion using lactic acid bacteria: Ginsenosides, GABA, and phenolic compounds. J. Microbiol. Biotechnol..

[5] Tsuchihashi R., Kodera M., Sakamoto S., Nakajima Y., Yamazaki T., Niiho Y., Nohara T., Kinjo J. (2009). Microbial transformation and bioactivation of isoflavones from Pueraria flowers by human intestinal bacterial strains. J. Nat. Med..

[6] Amaretti A., Raimondi S., Leonardi A., Quartieri A., Rossi M. (2015). Hydrolysis of the rutinose-conjugates flavonoids rutin and hesperidin by the gut microbiota and bifidobacteria. Nutrients.

[7] Di Cagno R., Mazzacane F., Rizzello C.G., Vincentini O., Silano M., Giuliani G., Angelis M.D., Gobbetti M. (2010). Synthesis of isoflavone aglycones and equol in soy milks fermented by food-related lactic acid bacteria and their effect on human intestinal Caco-2 cells. J. Agric. Food. Chem..

[8] Lloyd-Price J., Abu-Ali G., Huttenhower C. (2016). The healthy human microbiome. Genome Med..

[9] Duvallet C., Gibbons S.M., Gurry T., Irizarry R.A., Alm E.J. (2017). Meta-analysis of gut microbiome studies identifies disease-specific and shared responses. Nat. Commun..

[10] Ley R.E., Turnbaugh P.J., Klein S., Gordon J.I. (2006). Human gut microbes associated with obesity. Nature.

[11] Wang R., Tang R., Li B., Ma X., Schnabl B., Tilg H. (2021). Gut microbiome, liver immunology, and liver diseases. Cell. Mol. Immunol..

[12] Bonnechère B., Amin N., van Duijn C. (2022). The role of gut microbiota in neuropsychiatric diseases—Creation of an atlas-based on quantified evidence. Front. Cell. Infect. Microbiol..

[13] Carding S., Verbeke K., Vipond D.T., Corfe B.M., Owen L.J. (2015). Dysbiosis of the gut microbiota in disease. Microb. Ecol. Health Dis..

[14] DeGruttola A.K., Low D., Mizoguchi A., Mizoguchi E. (2016). Current understanding of dysbiosis in disease in human and animal models. Inflamm. Bowel Dis..

[15] Noda M., Maruyama M., Danshiitsoodol N., Higashikawa F., Sugiyama M. (2020). Improvement of alcohol-poisoning symptoms in mice by the oral administration of live *Lactobacillus plantarum* SN13T cells. Int. J. Mol. Sci..

[16] Gregory J.C., Buffa J.A., Org E., Wang Z., Levison B.S., Zhu W., Wagner M.A., Bennett B.J., Li L., DiDonato J.A. (2015). Transmission of atherosclerosis susceptibility with gut microbial transplantation. J. Biol. Chem..

[17] Cho C.E., Aardema N.D.J., Bunnell M.L., Larson D.P., Aguilar S.S., Bergeson J.R., Malysheva O.V., Caudill M.A., Lefevre M. (2020). Effect of choline forms and gut microbiota composition on trimethylamine-*N*-oxide response in healthy men. Nutrients.

[18] Garcia E., Shalaurova I., Matyus S.P., Wolak-Dinsmore J., Oskardmay D.N., Connelly M.A. (2022). Quantification of choline in serum and plasma using a clinical nuclear magnetic resonance analyzer. Clin. Chim. Acta.

[19] Brown J.M., Hazen S.L. (2015). The gut microbial endocrine organ: Bacterially derived signals driving cardiometabolic diseases. Annu. Rev. Med..

[20] Phimister E.G., Lynch S.V., Pedersen O. (2016). The human intestinal microbiome in health and disease. N. Engl. J. Med..

[21] Okamoto T., Sugimoto S., Noda M., Yokooji T., Danshiitsoodol N., Higashikawa F., Sugiyama M. (2020). Interleukin-8 release inhibitors generated by fermentation of *Artemisia princeps* Pampanini herb extract with *Lactobacillus plantarum* SN13T. Front. Microbiol..

[22] Noda M., Danshiitsoodol N., Inoue Y., Okamoto T., Sultana N., Sugiyama M. (2019). Antibiotic susceptibility of plant-derived lactic acid bacteria conferring health benefits to human. J. Antibiot..

[23] Panthavee W., Noda M., Danshiitsoodol N., Kumagai T., Sugiyama M. (2017). Characterization of exopolysaccharides produced by thermophilic lactic acid bacteria isolated from tropical fruits of Thailand. Biol. Pharm. Bull..

[24] Noda M., Miyauchi R., Danshiitsoodol N., Matoba Y., Kumagai T., Sugiyama M. (2018). Expression of genes involved in bacteriocin production and self-resistance in *Lactobacillus brevis* 174A is mediated by two regulatory proteins. Appl. Environ. Microbiol..

[25] Noda M., Kanno K., Danshiitsoodol N., Higashikawa F., Sugiyama M. (2021). Plant-derived *Lactobacillus paracasei* IJH-SONE68 improves chronic allergy status: A randomized, double-blind, placebo-controlled clinical trial. Nutrients.

[26] Noda M., Sugihara N., Sugimoto Y., Hayashi I., Sugimoto S., Danshiitsoodol N., Sugiyama M. (2021). *Lactobacillus reuteri* BM53-1 produces a compound that inhibits sticky glucan synthesis by *Streptococcus mutans*. Microorganisms.

[27] Noda M., Danshiitsoodol N., Sakaguchi T., Kanno K., Sugiyama M. (2021). Exopolysaccharide produced by plant-derived *Lactobacillus plantarum* SN35N exhibits antiviral activity. Biol. Pharm. Bull..

[28] Dyer W.J. (1945). Amines in fish muscle: I. colorimetric determination of trimethylamine as the picrate salt. J. Fish. Res. Bd. Canada.

[29] Hashimoto Y., Okauchi T. (1957). On the determination of TMA and TMAO: A modification of the Dyer method. Bull. Jpn. Soc. Sci. Fish.

[30] Caporaso J.G., Kuczynski J., Stombaugh J., Bittinger K., Bushman F.D., Costello E.K., Fierer N., Peña A.G., Goodrich J.K., Gordon J.I. (2010). QIIME allows analysis of high-throughput community sequencing data. Nat. Methods.

[31] Conn J.W., Rovner D.R., Cohen E.L. (1968). Licorice-induced pseudoaldosteronism, hypertension, hypokalemia, aldosteronopenia, and suppressed plasma renin activity. JAMA.

[32] Asl M.N., Hosseinzadeh H. (2008). Review of pharmacological effects of *Glycyrrhiza* sp. and its bioactive compounds. Phytother. Res..

[33] Zeisel S.H., Corbin K.D., Erdman J.W., MacDonald I.A., Zeisel S.H. (2012). Choline. Present Knowledge in Nutrition.

[34] Corbin K.D., Zeisel S.H. (2012). Choline metabolism provides novel insights into nonalcoholic fatty liver disease and its progression. Curr. Opin. Gastroenterol..

[35] Arias N., Arboleya S., Allison J., Kaliszewska A., Higarza S.G., Gueimonde M., Arias J.L. (2020). The relationship between choline bioavailability from diet, intestinal microbiota composition, and its modulation of human diseases. Nutrients.

[36] Romano K.A., Vivas E.I., Amador-Noguez D., Rey F.E. (2015). Intestinal microbiota composition modulates choline bioavailability from diet and accumulation of the proatherogenic metabolite trimethylamine-*N*-oxide. mBio.

[37] An X., Bao Q., Di S., Zhao Y., Zhao S., Zhang H., Lian F., Tong X. (2019). The interaction between the gut microbiota and herbal medicines. Biomed. Pharmacother..

[38] Hattori M. (1991). Metabolism of crude drug components by intestinal flora. Bifidus.

[39] Hattori M. (2012). Intestinal bacteria play a significant role in the medicinal effects of kampo medicines. J. Intest. Microbiol..

[40] Routy B., Le Chatelier E., Derosa L., Duong C.P.M., Alou M.T., Daillère R., Fluckiger A., Messaoudene M., Rauber C., Roberti M.P. (2018). Gut microbiome influences efficacy of PD-1-based immunotherapy against epithelial tumors. Science.

[41] Martinson J.N.V., Walk S.T. (2020). *Escherichia coli* residency in the gut of healthy human adults. EcoSal Plus.

[42] Krawczyk B., Wityk P., Gałęcka M., Michalik M. (2021). The many faces of *Enterococcus* spp.—Commensal, probiotic and opportunistic pathogen. Microorganisms.

[43] Ouwehand A.C., Salminen S., Isolauri E. (2002). Probiotics: An overview of beneficial effects. Antonie Leeuwenhoek.

[44] Marteau P., Seksik P., Lepage P., Dore J. (2012). Cellular and physiological effects of probiotics and prebiotics. Mini-Rev. Med. Chem..

[45] Bringel F. (1998). Carbamoylphosphate and natural auxotrophies in lactic acid bacteria. Lait.

[46] Li L., Chen B., Zhu R., Li R., Tian Y., Liu C., Jia Q., Wang L., Tang J., Zhao D. (2019). *. Fructus Ligustri Lucidi* preserves bone quality through the regulation of gut microbiota diversity, oxidative stress, TMAO and Sirt6 levels in aging mice. Aging.

[47] Horie M., Miura T., Hirakata S., Hosoyama A., Sugino S., Umeno A., Murotomi K., Yoshida Y., Koike T. (2017). Comparative analysis of the intestinal flora in type 2 diabetes and nondiabetic mice. Exp. Anim..

[48] Cerreto M., Santopaolo F., Gasbarrini A., Pompili M., Ponziani F.R. (2021). Bariatric surgery and liver disease: General considerations and role of the gut-liver axis. Nutrients.

[49] Guo W., Kim S.H., Wu D., Li L., Ortega E.F., Thomas M., Meydani S.N., Meydani M. (2021). Dietary fruit and vegetable supplementation suppresses diet-induced atherosclerosis in LDL receptor knockout mice. J. Nutr..

[50] Cassano M., Offner S., Planet E., Piersigilli A., Jang S.M., Henry H., Geuking M.B., Mooser C., McCoy K.D., Macpherson A.J. (2017). Polyphenic trait promotes liver cancer in a model of epigenetic instability in mice. Hepatology.

[51] Zeybel M., Arif M., Li X., Altay O., Yang H., Shi M., Akyildiz M., Saglam B., Gonenli M.G., Yigit B. (2022). Multiomics analysis reveals the impact of microbiota on host metabolism in hepatic steatosis. Adv. Sci..

[52] Loomba R., Seguritan V., Li W., Long T., Klitgord N., Bhatt A., Dulai P.S., Caussy C., Bettencourt R., Highlander S.K. (2017). Gut microbiome-based metagenomic signature for non-invasive detection of advanced fibrosis in human nonalcoholic fatty liver disease. Cell Metab..

[53] Del Chierico F., Nobili V., Vernocchi P., Russo A., De Stefanis C., Gnani D., Furlanello C., Zandonà A., Paci P., Capuani G. (2017). Gut microbiota profiling of pediatric nonalcoholic fatty liver disease and obese patients unveiled by an integrated meta-omics-based approach. Hepatology.

[54] Ahn S.B., Jun D.W., Kang B.K., Lim J.H., Lim S., Chung M.J. (2019). Randomized, double-blind, placebo-controlled study of a multispecies probiotic mixture in nonalcoholic fatty liver disease. Sci. Rep..

[55] Brahe L.K., Le Chatelier E., Prifti E., Pons N., Kennedy S., Hansen T., Pedersen O., Astrup A., Ehrlich S.D., Larsen L.H. (2015). Specific gut microbiota features and metabolic markers in postmenopausal women with obesity. Nutr. Diabetes.

[56] Lee G., You H.J., Bajaj J.S., Joo S.K., Yu J., Park S., Kang H., Park J.H., Kim J.H., Lee D.H. (2020). Distinct signatures of gut microbiome and metabolites associated with significant fibrosis in non-obese NAFLD. Nat. Commun..

